# Protein Prenylation in Plant Stress Responses

**DOI:** 10.3390/molecules24213906

**Published:** 2019-10-30

**Authors:** Michal Hála, Viktor Žárský

**Affiliations:** Department of Experimental Plant Biology, Faculty of Science, Charles University, Viničná 5, 128 44 Prague, Czech Republic; viktor.zarsky@natur.cuni.cz

**Keywords:** plants, stress, protein prenyl transferases, prenylated proteins

## Abstract

Protein prenylation is one of the most important posttranslational modifications of proteins. Prenylated proteins play important roles in different developmental processes as well as stress responses in plants as the addition of hydrophobic prenyl chains (mostly farnesyl or geranyl) allow otherwise hydrophilic proteins to operate as peripheral lipid membrane proteins. This review focuses on selected aspects connecting protein prenylation with plant responses to both abiotic and biotic stresses. It summarizes how changes in protein prenylation impact plant growth, deals with several families of proteins involved in stress response and highlights prominent regulatory importance of prenylated small GTPases and chaperons. Potential possibilities of these proteins to be applicable for biotechnologies are discussed.

## 1. Introduction to Protein Prenylation in Plants

Protein prenylation (i.e., addition of one or more isoprenoid side chains) is one of the key post-translational protein modifications which contributes significantly to the regulation of life processes at the lipid membrane-protein interface in most organisms. Such hydrophobic modifications permit otherwise hydrophilic proteins to operate as peripheral membrane proteins (for a general overview, see [1,2,3]).

Despite our focus on intensely studied protein prenylation in eukaryotes, it seems to be important for prokaryotic cells as well. This can be exemplified by the mutation of geranyl transferase IspA in *Staphylococcus aureus*, which causes reduced production of farnesyl pyrophosphate to one third of the wild type (WT) level, large changes in gene transcription, protein translation, decrease of the ATP level, and changes in cell envelope composition leading ultimately to a significantly higher sensitivity of mutant cells to oxidative stress [4].

The importance of protein prenyl modification differs also among eukaryotes. Only fifteen proteins, mostly RAB GTPases, were identified or predicted to be targets of prenylation in a large proteomic screen in *Plasmodium falciparum*, a protozoan malaria agent [5]. On the other hand, several hundred proteins are prenylated in multicellular animals and plants [1,2,3].

**Figure 1:**
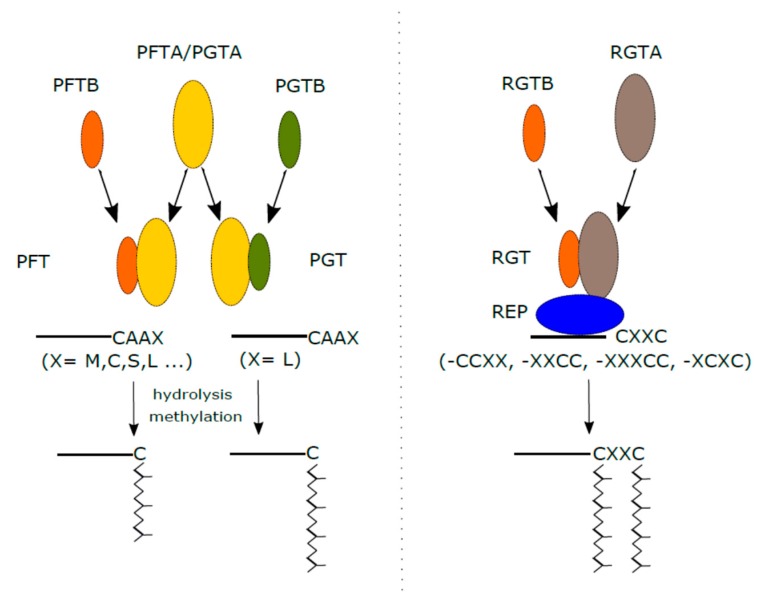
Protein prenylation in plants. The left side of the picture shows the structure of CAAX protein prenyl transferases (PFT and PGT), their preference for a C-terminal motif, processing of the prenylated protein, and a final form of the prenylated substrate. The right side shows the structure of RGT, involvement of the REP protein, possible C-terminal motifs, and a final form of the prenylated RAB GTPase.

Three different protein prenyl transferases catalyze the attachment of prenyl lipid anchors to the carboxyl termini of a variety of eukaryotic proteins (reviewed in [1,2]). Protein farnesyl transferase (PFT) catalyzes the posttranslational attachment of the farnesyl group (C15) via thioether linkage to a cysteine residue on the protein C-terminus. Protein geranylgeranyl transferase I (PGT) processes the geranylgeranyl group (C20) similarly. The third major type of protein prenyl transferases is Rab geranylgeranyl transferase (RGT), sometimes also called Protein geranylgeranyl transferase II (Figure 1). PFT and PGT are also called CaaX prenyl transferases because they recognize the carboxy- terminal -CaaX motif of substrate proteins, where “C“ stands for the cysteine residue and “a” stands for any aliphatic residue. The “X” residue determines the substrate specificity of CaaX prenyl transferases. Kinetic measurements for the recombinant *Arabidopsis thaliana* proteins reveal that PGT has highly specialized preference for CaaX motifs containing Leu on the last position, whereas PFT has a much broader specificity, including motifs terminating in Leu, although its affinity to these motifs was significantly lower than to motifs terminating in Met, Cys or Ser [6] (Figure 1).

CaaX protein prenyl transferases consist of two subunits, α and β (Figure 1). While β-subunits are encoded by transferase-specific genes, the α-subunit is shared by both enzymes [1]. CaaX prenyl transferases recognize only the carboxy-terminal CaaX motif of a substrate and the interaction between the cysteine residue and enzyme-bound Zn^2+^ is necessary for the proper transfer of the lipid anchor [7,8]. In all cases described, the prenylation is accompanied by further posttranslational protein processing involving hydrolytic cleavage of the –aaX group from the carboxy-terminal motif by specific proteases [9] and methylation of the free carboxyl group of the terminal cysteine residue [10]. The genome of *Arabidopsis* encodes two such proteases, FACE1/STE24 and FACE2/RCE1 and two methyltransferases (ICMT), which process prenylated proteins. Functional analysis of the two proteases revealed that both these proteins can complement mutation of their respective yeast homologues, Ste24p and Rce1p. Moreover, both proteases are localized exclusively on the endoplasmic reticulum. On the other hand, their (in vitro) substrate specificities differ based on their carboxy-terminal -CAAX motif composition [11]. Evolutionary conservation of all parts of the processing machinery, including methyltransferases, was further confirmed between plants and yeasts using complementation experiments [12].

In contrast to PFT and PGT, RGT does not require so specific carboxy- terminal motif [13] apart from the availability of several, mostly two, cysteine residues close to the carboxyl terminus. RGT seems to prenylate exclusively Rab GTPases, but it remains unclear whether this is the only enzyme geranylgeranylating Rab GTPases *in vivo* or if there is sharing of prenylation motifs with other protein prenyl transferases, since several members of the Rab family (e.g. Rab8) were shown to be prenylated also by PGT *in vitro* [14].

RGT also consists of two tightly associated α and β subunits. In contrast to other protein prenyl transferases, however, it does not recognize RAB GTPases directly, but only in a complex with Rab escort protein (REP) (Figure 1). The mechanism of RGT action differs from the other two CaaX prenyl transferases principally in several points. First, REP binds a newly translated RAB protein and forms a stable RAB-REP complex. Second, RGT is able to recognize only the RAB-REP complex as its protein substrate and catalyses the transfer of geranylgeranyl moieties to the relevant cysteine residues in two steps to achieve double prenylation [15]. After the geranylgeranylation reaction, RAB GTPase is delivered to a donor membrane compartment in the same manner as its regular cycling – i.e., in complex with the GDP dissociation inhibitor which is also evolutionary related to REP [15,16,17]. Informative structural and functional overview of plant RGTs is provided by [18].

### 1.1. Protein Farnesyl Transferase in Plant Stress Responses

The first evidence of PFT involvement in developmental processes, as well as stress responses, was brought about by the analysis of mutants with enhanced sensitivity and response to ABA. One of these mutants, *era1*, was later identified as loss of function (LOF) mutant in the β subunit of protein farnesyl transferase [19]. It was soon thereafter uncovered that its pleiotropic phenotype is partially caused by changes in floral meristem organization [20] accompanied by evidence that transcription factor APETALA1 is a target for farnesylation [21]. However, it took much longer to explain the phenotypic deviation manifested by enhanced response to ABA and stomatal closure. First, it was shown that the presence of ABA massively activates S-type anion channels in the mutant, leading to stomatal closure and enhanced resistance to drought stress [22]. Then, the identification of *era1* target protein site of action was narrowed down to somewhere between the signal perception and the calcium influx activation; *era1* guard cells quickly increased their calcium concentration in the presence of low ABA, resulting in the activation of S-type anion channel dependent current (which was, on the other side, close to WT level in the absence of exogenous ABA [23]). The same work showed the epistatic relation of *era1* to phosphatase 2C mutation *abi2* partially rescuing the ABA-insensitive phenotype of *abi2*.

Identification and sequence analysis of members of the ABA signalling transduction pathways revealed that there is no prenylation motif in these proteins, opening the possibility of an indirect influence [24,25]. Finally, two J2 and J3 members of the family HSP40 proteins (members of the heat shock protein family) were identified as targets of PFT and LOF mutation in both resulted in phenotypic deviation similar to *era-1* mutant [25]. Another prenylated protein CYP85A2, cytochrome P450, which catalyses the conversion of castasterone to brassinolide (one of the final steps in brassinosteroid biosynthesis) was also proposed to play a role in pleiotropic *era-1* phenotype development. The phenotype of *cyp85a2* mutant plants resembles that of *era1*. A mutated form of CYP85A2, lacking a prenylation motif on its C-terminus, failed to complement *cyp85a2* loss of function, indicating the importance of prenylation for its proper function [26]. It is necessary to mention, however, that the C-terminal cysteine-containing prenylation motif is not conserved in CYP85A2 orthologues across all plant species [26].

Along with increased drought resistance, *era1* plants differ in their reaction to other abiotic stresses – one of their main attributes being heat intolerance. Although *era1* plants were shown to be more resistant to acute short-term (40 min) temperature increase to 44 °C than WT, upon chronic long-term incubation at 37 °C, the viability of *era1* mutants dropped dramatically. Crossing with ABA-signalling mutants did not affect the phenotype, but crossing with *HSP101* chaperone *Arabidopsis* LOF mutant, which is overexpressed in *era1* mutant, removed the resistance to short term exposure to elevated temperature [27]. It seems possible that closed stomata do not allow sufficient transpiration and so cooling of the heat-stressed leaves.

### 1.2. Protein Geranylgeranyl Transferase I in Plant Stress Responses

The β-subunit of PGT is, unlike the α-subunit, unique for this complex. Its loss, however, is not accompanied by pronounced phenotypic deviations in higher plants. The *pgtb* mutants show enhanced response to exogenous ABA, similar to *era1,* but distinctly weaker, and almost normal stomatal closure in the absence of exogenous ABA [28]. Interestingly, in the moss *Physcomitrella patens*, the loss of the PGT β-subunit caused a more severe phenotypic deviation than loss of the PGT α-subunit, indicating different requirements and possibly prenylation specificities across plant species. While loss of PFT α-subunit function allowed the moss cells to maintain cell polarity and only cell growth rate was compromised, the loss of the PGT β-subunit resulted in complete loss of cell polarity characterized by rounded, chaotically dividing cells [29].

The α-subunit is common for both PGT and PFT and its loss in higher plants causes very severe phenotypic deviation characterized by homeotic changes in flowers, extremely enlarged meristems, and highly reduced growth of *pluripetala* (*plp*) plants [30]. The *plp* phenotype was combined with *era1* mutant phenotype and analysis shows no additive effect in the double mutant. Moreover, the double mutant was less sensitive to external ABA than *era1* plants themselves. It was recently shown that ROP2, one of plant homologues of Rho GTPases, remains localized to the cytoplasm of *plp* root hair trichoblasts and is degraded, which reduces the production of internal ROS and destabilizes overall microtubule organization [31].

### 1.3. Rab Geranylgeranyl Transferase

The RAB geranylation machinery is highly conserved across eukaryotes. Although the first characterized plant REP failed to complement yeast mrs6 mutation, only one amino acid change restored its activity in yeasts, demonstrating close functional homology of these proteins [16]. Unfortunately, all our attempts to isolate REP LOF mutants failed (our unpublished data and [32]). The possible lethality of such mutation is supported by the observation in the moss *P. patens*, where no knock-out lines could be prepared [29]. Similarly, no viable mutant was isolated for the α-subunit of RGT (our unpublished data and [29]).

In moss and several higher plants families including Brassicaceae, the RGTB subunit underwent independent duplication [29,33] and redundancy of the two paralogues allowed isolation of mutants. While the mutation of a single RGTB subunit surprisingly resulted in no phenotypic deviation in moss [29], a mutation in *Arabidopsis rgtb1* led to pleiotropic phenotypic deviations characterized by reduced plant growth, reduced apical dominance, delayed senescence, loss of shoot gravitropic response, compromised exo- and endocytosis, and a de-etiolated phenotype [33]. Later, it was shown that mutants produce pollen with a higher rate of aberrant phenotypes, and root hair growth is partially affected under *in vitro* cultivation. It was also shown that a double *rgtb1/rgtb2* mutation is pollen-lethal, resulting in shrunken pollen grains with disorganized endomembranes [34].

## 2. Substrates of Prenyl Transferases Involved in Abiotic Stress Responses

**Figure 2:**
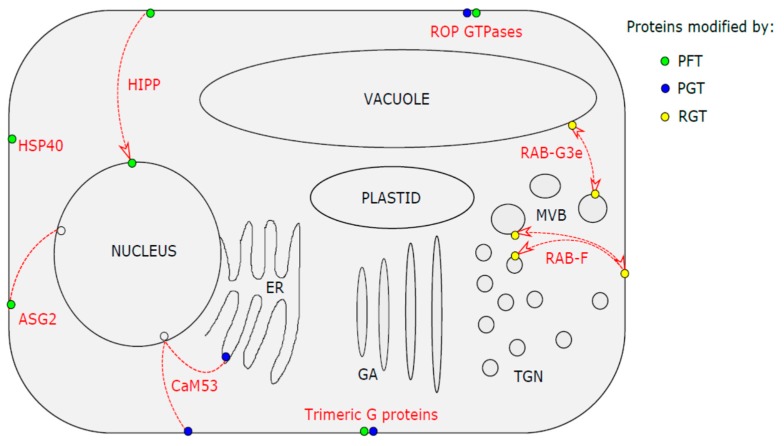
Graphical overview of sites of action relevant to protein groups involved in plant stress responses. Red lines connect localizations of different forms of proteins (also in red) as described in the text. When the direction of re-localization is applicable, it is marked by arrowheads. The colour of the circles corresponds to the modification reported (green – farnesylation by PFT, blue – geranylgeranylation by PGT, yellow – double geranylgeranylation by RGT, empty – non-prenylated). When two circles are present, both modifications were reported. ER- Endoplasmic reticulum, GA- Golgi apparatus, TGN- Trans-Golgi network, MVB- Multivesicular body.

In plants, many proteins are modified by prenylation and these proteins are important factors in many varied developmental processes, as well as in the acclimation of plants to their environment. In this review, we focus on the proteins involved in plant responses to stress (for reference to other types of processes, see the review by Running et al. [35]). The proteins focused on in this review are summarized in Table 1. For better orientation, Figure 2 provides a graphical overview describing sites of action for the groups of proteins listed, including types of isoprenoid modification.

### 2.1. Heat Shock Protein HSP40

Heat shock proteins are molecular chaperons that control and assist proteins conformation changes. As previously mentioned above, two members of the HSP40 family were shown to be prenylated and disruption of their genes lead to lethality in *Arabidopsis*. However, expression of their mutated form, lacking the Cys residue in the CaaX motif in double mutant background, restored viability but also resulted in functional changes in meristem organization and enhanced sensitivity to ABA, resembling *era-1* mutation, giving the mutant plants resistance to prolonged drought periods [25].

On the molecular level, strong up-regulation of members of the molecular chaperone family, namely HSP40, HSP70 and HSP90, were indeed observed also in *era1* mutants [25]. This work corresponds with older observation that the ANJ1 protein, identified as an *Atriplex nummularia* homologue of the bacterial DnaJ chaperone, is membrane localized in a farnesylated form; its expression in yeast can suppress the *mas5*, a yeast DnaJ homologue, mutation and restore growth in 37 °C [36].

Farnesylated HSP40 was also shown to co-immunoprecipitate with ARGONAUT1 (AGO1), a key member of the RISC complex that executes posttranscriptional mRNA level regulation. Although the impact of decreased a prenylation status of HSP40 on AGO1 localization is rather ambiguous, the decreased yield of HSP40 in CoIP experiments is clear. Moreover, intercellular gene silencing was partially compromised in farnesylation mutants, as roughly half of *era1* plants expressing a hairpin for silencing of magnesium chelatase subunit ChlI displayed the WT phenotype [55].

### 2.2. HIPP Proteins as Metallochaperons

The heavy metal-associated isoprenylated plant proteins (HIPP) family is a plant-specific family of many member proteins (e.g at least 44 paralogues in *Arabidopsis*) although not all of them are prenylated. Generally, HIPP proteins cluster, depending on author and phylogenetic analysis method used, into three [56] or seven [57] functionally distinct groups. They are involved in heavy metal homeostasis, drought and cold tolerance, and pathogen response [56,57].

#### 2.2.1. AtFPs

This family was one of the first isoprenylated metallochaperons groups described. Bioinformatic analysis has helped to describe genes encoding proteins capable of heavy metal- binding (an internal CXXC motif) and possessing the prenylation signal (a carboxy- terminal CaaX motif): three of these genes were found in *Arabidopsis*, and two in the soya (*Glycine max*) genome [37]. One of these proteins, Farnesylated protein 3 (AtFP3), was functionally characterized and its metal-binding capacity, as well as prenylation status, has been confirmed in vitro. Later, *Arabidopsis* Farnesylated protein 6 (AtFT6) was shown to be a plasma membrane bound protein, which was able to interact with an acyl-CoA binding protein 2 (ACBP2) and is also capable of binding transition metals. Its overexpression causes transgenic plants to have enhanced resistance to elevated Cd concentrations [38]. A similar effect was observed by overexpression of Cdl19, another metallochaperone containing two heavy metal- binding domains and prenylated on its C-terminus, which was found in the genome-wide screen for genes activated by cadmium [39]. Farnesylated metal-binding proteins were found also in barley (*Hordeum vulgare*) among stress-induced genes [40]. The transcript encoding the nuclear localized protein was shown to accumulate five hours after combined treatment with cold and high light as well as upon ABA treatment [40].

#### 2.2.2. HIPP26 and HIPP3

These proteins contain both - a heavy metal binding domain and a C-terminal prenylation motif and are localized to the nucleus or plasmatic membrane. Their proper localization on the membrane including nuclear envelope is dependent on prenylation. Expression of HIPP26 and its close homologues was studied under cold stress conditions. It was shown that the expression of these proteins differ at various time points. HIPP24 peaks in the late phase of cold stress, while HIPP23, HIPP25 and HIPP26 reach their maxima early on and their transcription is reduced in the later phase of the cold stress. HIPP26 itself is also induced by salt stress but does not respond to drought and heavy metals. It was also shown to interact with a zinc finger transcription factor ATHB29, which is known to mediate drought stress response in *Arabidopsis* [41].

Recently, HIPP26 was shown to be involved in long-distance transport of potato mop-top virus (PMTV) in *N. benthamiana*. The tobacco NbHIPP26 protein is localized to the plasmatic membrane and plasmodesmata due to the additional S-acylation and is, like its *Arabidopsis* homologue, expressed predominantly in vascular tissues. Infection by PMTV highly increases its expression, providing enhanced drought tolerance to the infected plant. Physical interaction with the virus releases NbHIPP26 from the membrane and the complex is delivered to the nucleus via microtubules [58].

HIPP3 is another metallochaperon expressed in *Arabidopsis*. Its expression increases upon pathogen attack, especially various *Pseudomonas* species. It is localized in the nucleus and affects the transcription of more than 400 genes involved in pathogen response [42].

### 2.3. Heterotrimeric G- Proteins

The heterotrimeric G- proteins are important factors involved in signalling in plant stress responses and prenylation of their subunits is necessary for function (see above). The morphological aspects of phenotypic deviations caused by mutations of particular G- protein subunits are thoroughly described in [59].

The γ- subunit was shown to be an important part of complex assembly and regulation of G- proteins function. Unlike α- (RGA) and β- (RGB) subunits, several γ- subunits (RGG) are encoded in single plant genomes. It was shown that two out of three rice homologues of RGG are differentially expressed under abiotic stresses. Moreover, RGG2 is a prominent factor responding to drought stress [43].

Trimeric G- proteins were shown to be involved in salt stress signalling in the shoot too, as loss of function of the *Arabidopsis* RGA subunit increased resistance to salt stress. Specifically, it caused delayed salt induced senescence, lower chlorophyll degradation, and cytoplasm leakage, although mutant plants contained the same concentration of salts as the WT [44]. Similarly, in rice, point mutated RGA subunit caused lower sensitivity to salt stress allowing a lower accumulation of ROS, although mutant plants displayed overall dwarf developmental phenotype [60]. Similarly, when subunits of mulberry trimeric G- proteins were ectopically overexpressed in tobacco, overexpression of RGA decreased the tolerance of transgenic tobacco plants to both drought and salinity stress while overexpression of other subunits had the opposite effect [61].

### 2.4. Prenylated Calmodulin and Ca^2+^ Stress Signalling

One petunia calmodulin, CaM53, was identified as a prenylation target. CaM53 is a substrate of PGT and is geranylgeranylated. The prenylated protein is localized at the plasmatic membrane while the protein with a mutated prenylation motif accumulates in the nucleus [45]. When the carboxymethylation is blocked, the protein is bound in the endomembrane system [62]. Overexpression of CaM53 in petunia plants resulted in stunted growth and necrosis, probably in connection with the disturbed Ca^2+^ signalling [62].

### 2.5. Protein Altered Seed Germination 2 (ASG2)

ASG2 was identified in a screen of cell cultures using covalently modified farnesyl supplemented to the cultivation media, which allowed for later proteomic identification of prenylated proteins. The ASG2 protein contains a WD40 repeat that was shown in later experiments to bind to the DDB1 protein, part of the ubiquitinylation machinery. The protein itself is nuclear-localized in its non- prenylated form. However, prenylation, especially farnesylation, prevents its nuclear localization. Mutants of ASG2 show enhanced response to external ABA and are more resistant to osmotic stress but, unlike *era1*mutants, water loss kinetics of detached leaves is similar to WT [46].

### 2.6. ROP GTPases in Plant Stress Response

ROP GTPases are small GTPases involved in many key cellular processes. Out of eleven *Arabidopsis* ROP GTPases, members of Class I contain C-terminal prenylation motif and were shown to be prenylated [63]. Developmental aspects of deficient ROP prenylation are described in [35].

Involvement of ROP GTPases in plant stress was studied in banana plants (*Musa acuminata*). Banana MaROPs were shown to be differentially expressed in salt stress conditions. Overexpression of MaROP5g in *Arabidopsis* plants caused increased salinity tolerance due to overexpression of SOS genes and a longer primary root [47].

Involvement of ROP GTPases in salinity stress tolerance was also observed in relation to the interaction of ROP2 and its interactor RIC1, inhibitor of microtubules reassembly. Reassembly of de-polymerized microtubules under salt stress conditions is important for the plant survival. Expression of RIC1 is therefore decreased during salt stress and interaction with ROP2 deactivates its inhibitory effect on microtubules reassembly. The phenotypic deviation of *rop2 Arabidopsis* mutants includes also salt sensitivity, which can be suppressed by *ric1* mutation [48].

Aside from its involvement in salt stress, involvement of ROP GTPases in ABA signalling during drought stress is also important. Already in the year 2002, analysis of *Arabdopsis rop10* mutants revealed a hypersensitive reaction to ABA in many aspects including stomatal closure - implying some ROP GTPases in the position of negative regulators of ABA response. Similarly, overexpression of constitutively active ROP10 GTPase decreased sensitivity to ABA. In both cases however, the level of ABA was not changed, neither was the reaction to other types of hormones [49]. Later, ROP11, a ROP member specific for guard cells of stomata, was shown to act similarly to ROP10, showing that ABA treatment causes nuclear localization of constitutively active proteins [64]. Similarly, overexpression of constitutively active RACB (a class I ROP GTPase) in barley caused higher water loss during osmotic stresses [50]. Stomatal conductance of transgenic leaves remained two times above WT level, even 16 hrs after leaf detachment, although their basal stomatal conductance under non-stress conditions was lower than the WT. Further experiments confirmed lower responsiveness of ROP transgenic plants to ABA treatment, which corroborates this phenomenon [50].

### 2.7. RAB GTPases in Plant Stress Response

RAB GTPases are molecular organizers of endomembrane trafficking. They possess low intrinsic GTPase activity (like most small GTPases) and are dependent in their cycling on several regulatory proteins, including GTPase activating proteins (GAPs), guanosine nucleotide exchange factors (GEFs), GDP dissociation inhibitors (GDIs) and GDI displacement factors (GDFs).

There are several RAB GTPases involved in the plant stress responses reported. One of them is *Arabidopsis* RAB-G3e, a RAB GTPase that is involved in endocytosis. Transcription of the RAB-G3e gene is elevated under oxidative stress and its transcription is induced by salicylic acid treatment. Salicylic acid is a crucial regulator involved in biotic stresses, thus it implies a possible involvement of RAB-G3e in biotic stress responses in plants. Overexpression of this RAB GTPase accelerated endocytosis and resulted in higher resistance to osmotic stresses for transgenic plants. When cultivated on media with a higher salt concentration, these transgenic plants had a higher salt content in their shoots when compared to the WT plants. This was correlated with increased salt accumulation in the vacuoles. Similar results were obtained when these plants were cultivated on media with a high sorbitol concentration. In this case, transgenic plants were also more resistant to osmotic stress, indicating that the effect was due to increased tolerance to the osmotic aspect of the salt stress rather than to ion toxicity [51].

The RAB-F subfamily of plant RAB GTPases (related to Rab5 of animals) is also involved in endocytosis regulation. The RAB-F1 member of this subfamily is unique, because it is the only known plant RAB that is not geranylgeranylated in a canonical manner and plays role in recycling materials from the endosomes to the plasmatic membrane, thus displaying both structural and functional differences from the RAB-F family [65]. Although it is acylated, it is worth mentioning in this review because it was shown to be essential in the stress response to elevated salinity [66]. Stunted root growth caused by increased salt or sorbitol concentrations (pointing clearly to an osmotic effect rather than salt toxicity) in the growth media could be overcome by overexpression of RAB-F1 protein. Conversely, plants lacking this protein were indistinguishable from WT plant [66]. High redundancy of RAB GTPases makes it difficult to study their individual roles in plant stress response. However, RAB-F1 was also shown to be involved in the build-up of an extrahaustorial membrane, the specific form of the plasmatic membrane surrounding fungal haustorium, protecting the plant cell against fungal invasion [67].

The involvement of the RAB-F subfamily in salt stress response was also documented in rice, *Oryza sativa*. Transgenic lines overexpressing OsRAB7, the RAB-F subfamily member, were shown to have significantly higher tolerance to salinity stress. This conclusion was based on the observation of a higher number of lateral roots and significantly faster shoot growth compared to non-transgenic controls, and accompanied by a significantly elevated level of proline accumulation under stress conditions as compared to the WT. Proline is a potent antioxidant and acts as a compatible solute for adjusting osmotic potential in the cytoplasm. Yet, the direct mechanism of how RAB GTPase induces proline accumulation is not known [52].

Data published about members of the exocytosis-involved RAB-A1 subfamily (related to Rab11 in animals) suggest that members of this subfamily are also required for tolerance to salinity stress. This implies that these RAB GTPases might regulate the localization of integral plasmatic membrane proteins, such as proton pumps and ion channels [68] or be directly involved in osmotic disposal to the apoplast or vacuole.

Indirect evidence for the involvement of RAB GTPases in plants coping with abiotic stress comes from studies on RAB regulatory proteins. Mutations in one of the nineteen GDI displacement factors (proteins that release RAB GTPases from GDIs and assist in their localization to donor membranes [69]) encoded in the *Arabidopsis* genome resulted in changes of vacuolar pH and higher sensitivity to salt stress [70].

## 3. Prenylated Proteins in Plant Biotic Stress Responses

Pathogen attack response is usually comprised of a large network of responsive genes. Describing these networks is out of the scope of this review, however, we will discuss some selected examples where prenylated proteins were directly shown to be involved in plant pathogen response.

ROP GTPases, previously described as important factors in plant response to abiotic stress, are also involved in biotic stress responses. It was shown that overexpression of a constitutively active form of class I ROP GTPase RACB in barley increased susceptibility to the plant fungal pathogen powdery mildew (*Blumeria graminis*), among other phenotypic deviations such as compromised shoot and root growth and decreased photosynthetic capacity. The effect was tightly connected with proper localization at the plasmatic membrane through its C-terminal prenylation [50]. It was shown that RACB GTPase, together with MLO-like proteins, induced changes in actin cytoskeleton in the pathogen attack area and transgenic cells were unable to reorganize the actin cytoskeleton under pathogen attack [71].

Transgenic *Arabidopsis* plants overexpressing dominant negative ROP6 displayed a pleiotropic developmental phenotype but also had elevated levels of salicylic acid, which made them more resistant to the powdery mildew pathogen. The effect of salicylic acid could be undone by creating a double mutants with salicylic acid production and or signalling defective mutants, but with no effect on other developmental aspects of the phenotype, confirming separation of these ROP roles [53].

Another group of prenylated proteins involved in plant-pathogen relations is represented by the *Arabidopsis* AIG1 protein family, also known as IAN8. Members of this family are highly inducible upon *Pseudomonas syringae* attack and contribute to plant immunity response as mutants are more susceptible to *Pseudomonas* infection [54].

## 4. Biotechnology Applications of Stress Prenylation Research in Plants

Protein prenylation can obviously be a promising target for applications in biomedicine and biotechnology [17]. First attempts were carried out with canola (*Brassica napus*) transformed with the antisense hairpin against the β- subunit of PFT under a drought-driven promoter. Under normal conditions, both WT and transgenic plants had the same amount of seeds, but at the onset of drought stress, especially during the season for flower and seed formation, the transgenic lines produced significantly more seeds [72].

Later, miRNA constructs were used under a drought inducible promoter of *Arabidopsis* hydroxypyruvate reductase to knock-down all four α subunits of PFT in *Brassica napus*. Although the level of mRNA was reduced to 50% of WT level in the best case, the seed yield was significantly better when the transgenic plants were exposed to limited water availability conditions. At the same time, the seed yield was as that of the WT when not exposed to water limitation [73].

As organizers of endomembrane trafficking, RAB GTPases are also potential targets for biotechnological manipulations. Investigations into the role of RAB GTPases in fruit ripening were initiated years ago. Fruit ripening is an economically important process related to significant cell wall modifications which result in softening of the whole fruit. RAB GTPases of the two subgroups- RAB-A and RAB-D - were shown to play a role in tomato (*Lycopersicon esculentum*) fruit ripening. Three paralogues of LeRAB1 (RAB-D subgroup) were detected, depending on the fruit development stage. While LeRAB1a was expressed in green growing fruits, the other two paralogues were highly expressed during the ripening phase [74]. Moreover, they seem not to be fully redundant in their function. Interesting data were also obtained for LeRAB11a (RAB-A subfamily). Transgenic tomato plants expressing antisense mRNA for *LeRAB11a* under the constitutive promoter produced fruits that changed color properly upon the ripening but remained firm for a long time (indicating the possibility for extended storage). The same authors also observed reduced activity of pectinesterase and polygalacturonase in the apoplast of these transgenic tomatoes [75]. Roles of RAB GTPases and other prenylated proteins in plant pathogen resistance have already been mentioned, and research focused on their function should not only bring new interesting discoveries in the near future, but also new biotechnology applications.

## Figures and Tables

**Table 1:** Isoprenylated proteins involved in plant stress responses.

Protein Name	Plant	Protein Accession	Stress	Reference
ANJ1	*Atriplex nummularia*	P43644	Abiotic – high temperature	[36]
HSP70 J2	*Arabidopsis thaliana*	NP_568412	Abiotic – high temperature, drought	[25]
HSP70 J3	*Arabidopsis thaliana*	Q94AW8	Abiotic – high temperature, drought	[25]
AtFP3	*Arabidopsis thaliana*	AAD09507	Abiotic – heavy metals	[37]
AtFP6	*Arabidopsis thaliana*	NP_195570	Abiotic – heavy metals	[38]
Cdl19	*Arabidopsis thaliana*	AAM64219	Abiotic – heavy metals	[39]
HvFP1	*Hordeum vulgare*	Q8GTD3	Abiotic – cold, strong light	[40]
AtHIPP26	*Arabidopsis thaliana*	OAP00180	Abiotic – cold, salinity	[41]
HIPP3	*Arabidopsis thaliana*	AIE40061	Biotic	[42]
RGG2	*Oryza sativa*	NP_001045833	Abiotic – drought	[43]
RGA1	*Oryza sativa*	ABF98475	Abiotic – salinity	[44]
CaM53	*Petunia x hybrida*	AAA33705	Abiotic	[45]
ASG2	*Arabidopsis thaliana*	OAO92260	Abiotic - salinity	[46]
MaROP5g	*Musa acuminata*	Ma09_p21130	Abiotic - salinity	[47]
ROP2	*Arabidopsis thaliana*	OAP19779	Abiotic - salinity	[48]
ROP10	*Arabidopsis thaliana*	OAP04715	Abiotic - drought	[49]
RACB	*Hordeum vulgare*	CAC83043	Abiotic - drought	[50]
RAB-G3e	*Arabidopsis thaliana*	NP_001031161	Abiotic – osmotic, Biotic	[51]
OsRAB7	*Oryza sativa*	AAO67728	Abiotic - salinity	[52]
ROP6	*Arabidopsis thaliana*	OAP00270	Biotic	[53]
AIG1	*Arabidopsis thaliana*	P54120	Biotic	[54]
